# Dihydrocapsiate does not increase energy expenditure nor fat oxidation during aerobic exercise in men with overweight/obesity: a randomized, triple-blinded, placebo-controlled, crossover trial

**DOI:** 10.1080/15502783.2022.2099757

**Published:** 2022-07-19

**Authors:** Francisco J. Osuna-Prieto, Francisco M. Acosta, Unai A. Perez de Arrilucea Le Floc’h, Blanca Riquelme-Gallego, Elisa Merchan-Ramirez, Huiwen Xu, Juan Carlos De La Cruz-Márquez, Francisco J. Amaro-Gahete, Jose A. Llamas-Elvira, Eva M. Triviño-Ibáñez, Antonio Segura-Carretero, Jonatan R Ruiz

**Affiliations:** a Department of Physical and Sports Education, Faculty of Sports Science, PROFITH “PRO-moting FITness and Health Through Physical Activity” Research Group, Sport and Health University Research Institute (iMUDS), University of Granada, Granada, Spain; b Department of Analytical Chemistry, University of Granada, Granada, Spain; c Research and Development of Functional Food Center (CIDAF), Granada, Spain; d Turku PET Centre, University of Turku, Turku, Finland; e Turku PET Centre, Turku University Hospital, Turku, Finland; f Department of Nursing, Faculty of Health Sciences, University of Granada, Granada, Spain; g Instituto de Investigación Biosanitaria de Granada (ibs.GRANADA), Granada, Spain; h CIBER of Epidemiology and Public Health (CIBERESP), Carlos III Institute of Health, Madrid, Spain; i Department of Physiology, Faculty of Medicine, EFFECTS-262 Research group, University of Granada, Granada, Spain; j Department of Nuclear Medicine. Hospital Universitario Virgen de las Nieves, Granada, Spain

**Keywords:** Capsinoids, TRPV1, obesity, nutraceutical, FATmax, metabolism

## Abstract

**Background:**

Prior evidence suggests that capsinoids ingestion may increase resting energy expenditure (EE) and fat oxidation (FATox), yet whether they can modulate those parameters during exercise conditions remains poorly understood. We hypothesized that dihydrocapsiate (DHC) ingestion would increase EE and specifically FATox during an acute bout of aerobic exercise at FATmax intensity (the intensity that elicits maximal fat oxidation during exercise [MFO]) in men with overweight/obesity. Since FATmax and MFO during aerobic exercise appear to be indicators of metabolic flexibility, whether DHC has an impact on FATox in this type of population is of clinical interest.

**Methods:**

A total of 24 sedentary men (age = 40.2 ± 9.2 years-old; body mass index = 31.6 ± 4.5 kg/m^2^ [n = 11 overweight, n = 13 obese]) participated in this randomized, triple-blinded, placebo-controlled, crossover trial (registered under ClinicalTrials.gov Identifier no. NCT05156697). On the first day, participants underwent a submaximal exercise test on a cycle ergometer to determine their MFO and FATmax intensity during exercise. After 72 hours had elapsed, the participants returned on 2 further days (≥ 72 hours apart) and performed a 60 min steady-state exercise bout (i.e. cycling at their FATmax, constant intensity) after ingesting either 12 mg of DHC or placebo; these conditions were randomized. Respiratory gas exchange was monitored by indirect calorimetry. Serum marker concentrations (i.e. glucose, triglycerides, non-esterified fatty acids (NEFAs), skin temperature, thermal perception, heart rate, and perceived fatigue) were assessed.

**Results:**

There were no significant differences (P > 0.05) between DHC and placebo conditions in the EE and FATox during exercise. Similarly, no significant changes were observed in glucose, triglycerides, or NEFAs serum levels, neither in the skin temperature nor thermal perception across conditions. Heart rate and perceived fatigue did not differ between conditions.

**Conclusions:**

DHC supplementation does not affect energy metabolism during exercise in men with overweight/obesity.

## 1. Introduction

Poor nutritional habits, sedentarism, and physical inactivity are among the foremost modifiable risk factors related to cardiovascular disease (CVD) and all-cause mortality [1]. Optimizing any of these components is key to improving cardiometabolic health and weight loss success in the long term [2]. In this regard, the use of nutraceuticals and natural food ingredients aiming to increase energy expenditure (EE) and fat oxidation (FATox) has attracted a great deal of attention over the past decade, especially among overweight/obese individuals [3].

Capsaicinoids are a family of pungent compounds that are found in chili peppers and other spicy foods. Ingestion of capsaicin, the most abundant capsaicinoid, has been shown to elicit significant increases in EE and FATox in humans [4]. Nonetheless, tolerance to capsaicin varies between individuals, and its ingestion in high doses could lead to pain, swelling, and gastrointestinal problems [5]. Capsinoids, which include capsiate, dihydrocapsiate (DHC), and nordihydrocapsiate, are significantly less pungent chemical analogs of capsaicin [6]. The ingestion of capsaicin and capsinoids activates the transient receptor potential vanilloid subfamily member 1 (TRPV1) in the gastrointestinal tract [7]. In fact, the ingestion of CH-19 sweet pepper, a capsinoid-rich variety of pepper, increases core body temperature [8,9] and oxygen consumption [8], which is likely to be explained by increased sympathetic nervous activity and catecholamines levels [9,10]. The use of purified capsinoids has been suggested to yield similar effects, although this needs to be further examined [11–13].

Given that endurance training programs effectively help in weight loss and maintenance, and in cardiometabolic risk management in adults with overweight/obesity [14], the use of capsinoids as potential coadjutants to increase EE or FATox during exercise is of particular interest. Thus, far, only two studies have investigated the effect of capsinoids (i.e. capsinoids mixture [capsiate, DHC, and nordihydrocapsiate] or capsiate) ingestion on EE and FATox during aerobic exercise in healthy lean active young men, showing a lack of effect [15,16]. Importantly, these studies were conducted on normal-weight subjects. However, one previous study has also shown that capsinoids intake may enhance EE and FATox, particularly in individuals with overweight/obesity in resting conditions [17]. Therefore, we hypothesized that ingestion of DHC, an isolated capsinoid type currently available in the market – and whose effects have been tested only in resting conditions [11,18] – would further increase the EE and FATox during an exercise bout designed to elicit maximal fat oxidation (MFO) in sedentary men with overweight/obesity. Based on previous studies using CH-19 sweet red peppers that indicate that the metabolic effects of capsinoids take place within the first 10 minutes after ingestion and last for at least 60 minutes [8,9], our study was designed to evaluate the DHC effects during this specific frame time. Since the previous DHC studies employed doses of up to 9 mg of DHC [11,18], we decided to administrate 12 mg of DHC (the highest dose approved by the European Food Safety Authority (EFSA)) to ensure its effectiveness.

The aim of the present study was to investigate the acute effects of the ingestion of DHC on EE and FATox during a bout of 60 min of aerobic exercise at FATmax intensity in men with overweight/obesity. As a secondary aim, we investigated the effects of DHC on other physiological parameters, such as blood markers, skin temperature, thermal perception, heart rate, and perceived fatigue.

## 2. Material and methods

### Study subjects and experimental design

The current work was conducted within the framework of the ACTIFOX (ACTIvating Fat OXidation through capsinoids) study, a randomized, triple-blinded, placebo-controlled, crossover trial designed to determine the effect of DHC on EE and FATox during aerobic exercise (ClinicalTrials.gov ID: NCT05156697). A total of 24 men with overweight or obesity (40.2 ± 9.2 years old, body mass index (BMI) >25 kg/m^2^) participated in the study. The caption flow of the participants in the ACTIFOX study is depicted in **Fig. S1**. Inclusion criteria were to be male and 18–55 years old, to be sedentary (subjects reported <20 min moderate to vigorous physical activity on <3 days/week), to be nonsmoker, not being under medication that could affect energy metabolism, and to have a stable body weight over the preceding 3 months (<3 kg change). Exclusion criteria were having been diagnosed with diabetes, hypertension, or any medical or cardiometabolic condition(s) that could interfere with or be aggravated by exercise, presenting a family history of CVD, having an abnormal electrocardiogram, regular and high consumption of spicy foods, and being frequently exposed to cold temperatures (*e.g*. indoors/outdoors workspace with low-temperatures, such as cold-storage works, ski/snow monitors, etc.). All participants gave their written informed consent. The study protocol and design were approved by the Human Research Ethics Committee of the University of Granada (n°839/CEIH/2019) and the Servicio Andaluz de Salud and adhered to the tenets of the Declaration of Helsinki as revised in 2013. The study was carried out in Granada (Spain), from October 2019 to March 2020.

## 3. Procedures

### Overall procedures

**Fig. S2** shows the overall design of the study. Data for each participant were collected over 4 visits to the research center within 3 weeks. Participants were asked to confirm having commuted to the research center by car, bus, tram, or motorcycle, having slept as usual, having refrained from stimulant beverages within 24 h, and having avoided any moderate or vigorous physical activity within 24 h and 48 h (respectively). Other specific pre-experimental conditions were established for each visit are detailed in the following sections.

Briefly, in the first visit, sociodemographic and lifestyle data were registered, a medical screening was performed, blood samples were collected, and anthropometry and body composition measures were taken. On the second visit, the MFO during exercise and cardiorespiratory fitness (VO_2_ peak) were, respectively, assessed through a submaximal exercise test coupled to a maximal effort test. On visits third and fourth, participants performed a 60 min steady-state exercise bout on a cycle ergometer at FATmax intensity (i.e. at the intensity at which MFO is elicited) after having ingested either 12 mg of DHC or placebo. The conditions (DHC or placebo) on visits 3 and 4 were randomized. The washout period between visits 3 and 4 was ≥72 h. Of note, all exercise tests took place at a strictly controlled temperature of 22-23°C, given that environmental temperature largely influences EE and FATox [19].

### Medical screening, sociodemographic data collection, and anthropometry and body composition assessments

On the first visit, participants arrived at the research center at 08:00 h, in fasting conditions (8 h). They were informed about the study protocols and gave their oral and written informed consent to participate in the study. Sociodemographic data and details related to their dietary habits (including pungent consumption), appetite, physical activity levels, sleep, and other lifestyle habits were recorded by questionnaires. Dietary intake was assessed using a previously validated food frequency questionnaire (FFQ) and three 24 h-recalls undertaken on three nonconsecutive days, as previously described [20]. Afterward, a medical doctor conducted an anamnesis to ensure that each participant was in suitable physical conditions to participate in the study and to perform the exercise. Subsequently, participants underwent an electrocardiogram in resting conditions conducted by an expert medical doctor. Systolic and diastolic blood pressure were also measured with an automatic sphygmomanometer (Omron M2; Omron Healthcare, Kyoto, Japan). Measurements were repeated on 3 consecutive occasions, and the average systolic and diastolic blood pressure were calculated. Only participants presenting a non-risk medical history and normal electrocardiogram were allowed to participate in the study.

Blood samples were obtained from the antecubital vein in the morning (8.00–9.00 am), with subjects sitting and in resting conditions. Blood samples were collected in serum Vacutainer Tubes® (Vacutainer® SST™ II Advance tubes) and centrifuged following the manufacturer’s instructions. Afterward, serum samples were sent to the hospital lab for the analysis of the analytes of interest.

Anthropometry and body composition assessments also took place on the first visit. Bodyweight and height were measured (no shoes, light clothing) using a model 799 Seca scale and stadiometer (Seca, Hamburg, Germany). Waist circumference was assessed twice at the minimum perimeter area with a measuring tape (mm precision), and the mean value was calculated. For those participants with abdominal obesity, waist circumference was measured just above the umbilicus (horizontal plane). Body fat mass and percentage, lean body mass, and visceral adipose tissue (VAT) mass were then measured by whole-body dual-energy X-ray absorptiometry (HOLOGIC, Discovery Wi, Marlborough, MA). Body mass, lean mass, and fat mass indexes were calculated as kg/m^2^.

### Exercise tests

On the second visit, individuals arrived at the research center, between 15:30 and 19:00. Participants confirmed having met the above-stated pre-experimental conditions, as well as arriving in fasting conditions (5–6 h) and having carried out a standardized diet that they were instructed to follow during the previous day. Then, they emptied their bladders, dressed in standardized t-shirts and shorts, and entered a quiet, warm (22-23°C) room. A submaximal-graded exercise test (to determine the MFO) plus a maximum effort test (used to reach the VO_2_peak) was performed employing an Ergoselect 200 cycle ergometer (Ergoline GmbH, Lindenstrasse, Germany). The exercise protocol, coupled to indirect calorimetry, started with a 3 min stage at 20 watts (W) as a warm-up, followed by increments of 20 W every 3 min, until the respiratory exchange ratio (RER) was ≥1 at least for 30s (as determined by indirect calorimetry) [21,22]. At this point, the maximal exercise protocol started (with no interruptions), implementing further increments of 20 W every 1 min until (i) volitional exhaustion was reached, or (ii) participants had to stop because of peripheral fatigue. The participants were asked at the end of the test whether volitional exhaustion or peripheral fatigue forced them to stop the exercise test. Of note, the cycling power values (W) at which MFO happened for each individual were used as the target exercise intensity (i.e. FATmax) for the subsequent steady-state tests. Through the exercise test, participants’ perceived fatigue was assessed using a rating of perceived exertion (RPE) scale, and heart rate was measured using a Polar RS800 heart-rate monitor (Polar Electro Inc., Woodbury, NY, USA). Respiratory gas exchange was monitored with a CPX Ultima CardioO2 system (Medical Graphics Corp., St Paul, MN) with a facemask model 7400 (Hans Rudolph Inc., Kansas City, MO), and a preVent™ metabolic flow sensor (Medical Graphics Corp.) [23]. Oxygen consumption (VO_2_) was measured using a galvanic fuel cell and carbon dioxide production (VCO_2_) was assessed using a non-dispersive infrared sensor [23]. According to the manufacturer’s recommendations, the gas analyzer was calibrated using standard gas concentrations immediately before each test. Of note, an experienced medical doctor continuously monitored the heart rhythm and electrical activity through the whole exercise test by means of an electrocardiogram, with the test stopped if required by the doctor based on medical criteria.

### Steady-state exercise bout

On the third visit (≥72 h after the second day), and on the fourth visit (≥72 h later after the third visit to avoid carry-out effects), participants came to the laboratory and underwent the steady-state tests after the ingestion of DHC or placebo, in a randomized order. Participants arrived at the same time as on the second visit and confirmed having met the same pre-experimental conditions. **Figure S3** shows the design of the steady-state exercise tests. Participants urinated, dressed in standard clothing, and entered a quiet, warm (22-23°C) room. A Polar RS800 heart-rate monitor was placed on their chest using a chest wrap band. Then, a set of 16 DS-1922 L iButton^TM^ wireless thermometers (Thermochron, Dallas, TX, USA) were attached to the subject’s skin in different places to monitor skin temperature changes throughout the experiment. They were put on the *forehead, left pectoralis, left elbow region, left index fingertip, left forearm, rear neck central area, right clavicula, right deltoid, right shinbone, right sub-clavicular area, right supra-clavicular area, right thigh, and upper breastbone*. Afterward, participants sat and stay relaxed for 10 min (resting period, timepoint −20´), a time during which they were instructed not to move nor cross their arms and legs, and their baseline skin temperature and heart rate measures were taken. The first intravenous blood sample was collected 10 min before starting the steady-state test (timepoint −10’). Immediately 3 min after the first blood collection (timepoint −7’), participants ingested either 12 mg of DHC (4 pills 3 mg each one) or placebo. Then, they sat on the cycle ergometer where the steady-state tests would be performed, and they put on their faces a gas mask for the gas exchange measurement. The same metabolic cart as on the second visit was used. The gas collection started 1 min before the beginning of the steady-state test (timepoint −1’) with the participants sitting on the cycle ergometer without pedaling. After 1 min of gases recording in resting conditions, the steady-state test at FATmax intensity started and continued (constant intensity) until the minute 60, the moment at which the test finished. Gases exchange and heart rate were continuously monitored. At time points 15, 30, 45, and 60 min, blood samples were collected to determine serum levels of glucose, triglycerides, and NEFAs. Simultaneously, participants completed the ASHRAE scale to record their thermal perception. Every 5 min, participants were asked to report their fatigue perception using RPE scales.

### Test substances: dihydrocapsiate and placebo

We employed Capsiate Gold™ soft-gel capsules from Ajinomoto® (Ajinomoto Health & Nutrition North America, Inc., JP). These capsules consisted of 3 mg of purified DHC vehiculated with canola oil, modified corn starch, vegetable glycerin, carrageenan, water, disodium hydrogen phosphate, and soy lecithin. Microcrystalline cellulose powder (Fagron Ibérica, Terrassa, SP) was used as placebo. Both DHC and hemicellulose were encapsulated by independent manufacturers and put in different containers by an independent researcher (not involved in the current study). Each container was labeled with a different code (0 or 1) and, thus, evaluators were not aware of the administered substance – therefore preventing bias. Of note, both DHC and placebo capsules looked exactly similar to unable the identification of the content by either researchers or participants.

## 4. Outcomes

### MFO, FATmax, and cardiorespiratory fitness

Gas exchange data were obtained and exported from the metabolic carts Breeze Suite (8.1.0.54 SP7) software (MGC Diagnostics Corp.) to Excel for Windows. During the submaximal exercise test, VO_2_ and VCO_2_ data were averaged over the last 60s of each 3 min stage [24], and FATox was estimated from these values by using Frayn stoichiometric equations [25] (shown below; urinary nitrogen excretion was assumed to be negligible) [26]. The obtained FATox values (g/min) from the different stages of the submaximal exercise test were plotted against the relative exercise intensity (W). Third-degree polynomial regression was subsequently built to determine the absolute maximal fat oxidation (MFO, g/min). FATmax was calculated as a function of VO_2_peak (i.e. % VO_2_peak) by selecting the VO_2_ value at the temporal moment at which MFO was elicited, and plotting this value against the estimated VO_2_peak, expressing it as a percentage. Maximal VO_2_ (VO_2_max) was defined as a respiratory exchange ratio of ≥1.1, once a VO_2_ plateau was reached and having attained a heart rate value within 10 beats/min of the individuals’ age-predicted maximum (209-0.73× age) [27] during the maximal exercise test. However, participants did not achieve the VO_2_max criteria, and therefore VO_2_ peak was determined as the highest VO_2_ value that was not an artifact. This value was provided relative to the body mass.

### Gases exchange parameters during steady-state tests

Gas exchange data were downloaded and averaged every 1 min as stated above. Then, VO_2_ and VCO_2_ for each selected data point were used to estimate EE, RER, and substrate oxidation rates – comprised of carbohydrate oxidation (CHOox) and FATox. EE was estimated using Weir’s abbreviated equation [28]. Frayn’s stoichiometric equations [25] were used for estimating the CHOox and FATox. Urinary nitrogen excretion was assumed to be negligible and therefore was not included in the formula [29].

$$Energy\;\;\;expenditure\;\;\;\left(Kcal/min\right)=(1.106*VCO_{2})+(3.941*VO_{2})$$

$$RER=\left(VCO_{2}/VO_{2}\right)$$

$$CHOox\;\left(g/min\right)=(4.55*VCO_{2})-(3.21*VO_{2})$$

$$FATox\;\left(g/min\right)=(1.67*VO_{2})-1.67*VCO_{2})$$

To calculate the EE variables that were used in the analyses, the 60 min-duration steady stage was split into 5 min stages, and the average EE of each stage was calculated. Therefore, a total of 12 mean EE values (one per stage) were obtained. These values were used for the analyses examining the mean values at each time point of EE. Next, the area under the curve (AUC, trapezoidal rule) and the AUC expressing it as a percentage of its baseline – AUC (% baseline) – were calculated. The same procedure was followed to calculate the RER, CHOox, and FATox during the steady-state test. The obtained parameters were used in the subsequent analyses.

### Serum parameters

Serum glucose, non-esterified fatty acids (NEFAs), total cholesterol (TC), high-density lipoprotein-cholesterol (HDL-C), triglycerides (TG), and liver enzymes (alkaline phosphatase [ALP], gamma-glutamyl-transferase [GGT], and glutamate-pyruvate transaminase [GPT]) were assessed following standard methods using an AU5832 automated analyzer (Beckman Coulter Inc., Brea, CA, USA). Low-density lipoprotein-cholesterol (LDL-C) was estimated as [TC – HDL-C – (TG/5)], with all units expressed in mg/dL [30]. Serum insulin was measured using the Access Ultrasensitive Insulin chemiluminescent immunoassay kit (Beckman Coulter Inc., Brea, CA, USA). The homeostatic model assessment for insulin resistance index (HOMA-Index) was calculated as [insulin (μU/mL) × glucose (mmol/L)/22.5 [31]], whereas fatty liver index was calculated using a commonly used procedure [32]. C-reactive protein was measured by an immunoturbidimetric assay using an AU5832 automated analyzer (Beckman Coulter Inc., Brea, CA, USA).

### Skin temperature

A total of 16 iButtons® were attached to the skin in different spots (as previously explained). Skin temperature measurements during the steady-state were taken every 60s using DS-1922 L Thermochron iButtons® (resolution: 0.0625 ^◦^C) (Maxim, Dallas, USA) [33]. The iButtons® programming, as well as the downloading and pre-processing of raw data were conducted using Temperatus® software [34]. After conducting the experiment, data were downloaded every 60s for each iButton, in a CSV file. Atypical data were eliminated by suppressing the time points for which the rate of change with respect to the previous value was higher than the interquartile distance between quartiles 1 and 3 for all data (percentiles 25 and 75, respectively [35];). Then, these data were divided into blocks of 5 min, and their average was calculated, obtaining 12 mean values (one for each 5 min-block of the 60 min steady-state test). Finally, the overall mean [36], proximal [37], and distal skin temperatures were calculated using the Temperatus® software – see references [19,38] for further information. The validity and reliability of this system have been established for the assessment of skin temperature in humans [35,39]. The equations used (see below) have been described elsewhere [38].

*Overall mean skin temperature* = (Forehead*0.07) + (Right Scapula*0.175) + (Left Chest*0.175) + (Right Deltoid*0.07) + (Left Elbow*0.07) + (Left Hand*0.05) + (Right Thigh*0.19) + (Right Gastrocnemius*0.2).

*Proximal skin temperature* = (Right Thigh*0.383) + (Right Clavicular*0.293) + (Right Abdomen*0.324).

*Distal skin temperature* = (Left Hand+Right Instep)/2

### Thermal perception

Thermal perception was assessed using the scale from the American Society of Heating, Refrigerating and Air Conditioning Engineers (ASHRAE), which is composed of 7 items in which subjects are asked about their thermal perception over the whole body and different body regions (the clavicular and abdominal regions, arms, hands, legs, and feet). Each scale’s item ranges from cold (−3), cool (−2), slightly cool (−1), neutral (0), slightly warm (1), warm (2), to hot (3). Shivering perception was measured using a numerical scale that ranges from 0 to 10, where 0 means ‘I am not shivering’ and 10 means “I am shivering a lot.”

### Sample size

Based on previous studies [40], a total of 12 participants per group would be needed to be able of establishing statistical differences between conditions (placebo vs. DHC) in EE (~10%) and FATox (~10–15%) in resting conditions (80% statistical power; α = 0.05). Since this is a cross-over study – each participant serves as its own control – the minimum of participants required is n = 12.

### Randomization

Participants ingested DHC or placebo prior to the steady-state test in a randomized order. This randomization was performed with Excel’s Data Randomizer Function (without blocking for any variable or imposing restrictions) by FJAG – who did not participate in the assessments nor in the experiments.

### Blinding

The triple-blinding design consisted of: (i) an independent third researcher (not directly involved in this study) conducted the encapsulation of both DHC and placebo in standard pills to unable the identification of the substance by either participants or researchers. These pills were named condition 1 or condition 2 pills; (ii) none of the researchers involved in the experimental phases and assessments knew whether the condition 1 or 2 corresponded to DHC or placebo, and neither did the participants; (iii) during the data and statistical analyses, no one of the team members was aware of the content of conditions 1 and 2, except FAM, who was the coordinator of data analysis (i.e. data analysis was also blinded); (iv) only when all the statistical analyses were conducted and finalized by FJOP, the content of condition 1 and 2 pills was unveiled.

### Statistical analysis

Descriptive statistics of the study subjects are shown as mean ± standard deviation. Data normality was assessed using the Shapiro–Wilk test, histograms, and Q-Q plots. The parameters that did not follow a normal distribution (i.e. gases parameters) were log10-transformed to achieve normal data distribution. Gases exchange parameters during aerobic exercise in the DHC vs. placebo condition were compared using a T-test for paired samples. Linear mixed model analyses were used to examine the gas exchange parameters during exercise after DHC or placebo ingestion. These analyses (i.e. paired T-tests and linear mixed models) were replicated with the serum analytes, skin temperature, and thermal perception across conditions. The level of significance was set at P < 0.05. The statistical analyses were performed using the Statistical Package for the Social Sciences v.26.0 (IBM Corporation, Chicago, IL, USA). GraphPad Prism version 8.0.0 (GraphPad Software, San Diego, CA) was used to plot the figures including respiratory exchange and blood parameters.

## 5. Results

As shown in **Figure S1**, from the 32 subjects who were recruited from October 2019 to February 2020 and completed the basal assessment and fulfilled the conditions after the medical check (i.e. they met the inclusion criteria and were therefore enrolled), 5 of them refused to continue because of problems with adhering to the schedule and experimental conditions. The other 3 subjects were excluded after being randomized and undergoing the steady-state test, due to problems or abnormalities during these tests. Therefore, a final sample size of n = 24 was included for our main analyses, having all participants valid and complete gases exchange data collection. The sample size varied for the measures related to the secondary aim. The characteristics of the participants are shown in Table 1.

**Table 1.** Descriptive data of the study subjects (n = 24).

	N	Mean		SD
Age (years)	24	40		9
*Anthropometry and body composition*				
Body mass index (kg/m^2^)	24	31.6	±	4.5
Waist circumference (cm)	24	107.1	±	11.1
Lean body mass (kg)	24	58.1	±	6.6
Lean mass index (kg/m^2^)	24	19.0	±	2.3
Fat mass (kg)	24	33.7	±	8.6
Fat mass index (kg/m^2^)	24	11.0	±	2.7
Body fat percentage (%)	24	35.3	±	4.9
Visceral adipose tissue mass (g)	24	818	±	320
*Fasting cardiometabolic profile*				
Glucose (mg/dL)	23	94	±	8
Insulin (μIU/mL)	23	11	±	6
HOMA-index	22	2.6	±	1.4
GTP (IU/L)	23	33	±	15
GGT (IU/L)	23	42	±	29
ALP (IU/L)	23	72	±	21
Total cholesterol (mg/dL)	23	201	±	28
HDL-C (mg/dL)	22	48	±	9
LDL-C (mg/dL)	22	130	±	21
Triglycerides (mg/dL)	23	129	±	51
Systolic blood pressure (mmHg)	24	126	±	14
Diastolic blood pressure (mmHg)	24	86	±	11
*MFO, FATmax and cardiorespiratory fitness*				
MFO (g/min)	24	0.24	±	0.09
MFO/LM (mg/kg/min)	24	4.05	±	1.43
FATmax (%VO_2_peak)	24	33	±	7
VO_2_peak (mL/min)	24	2845	±	473
VO_2_peak/lean mass (mL/kg/min)	24	30	±	6

Data are presented as mean and standard deviation (SD). ALP, alanine phosphatase, GGT: gamma-glutamyl transferase, GPT: glutamate-pyruvate transaminase, HDL-C: High-density lipoprotein-cholesterol, HOMA: homeostatic model assessment, LDL-C: Low-density lipoprotein-cholesterol, LM: lean mass, MFO: maximal fat oxidation, VO_2_: volume of oxygen.

### Dihydrocapsiate effects on energy expenditure and fat oxidation during aerobic exercise at FATmax intensity

**Figure 1.**
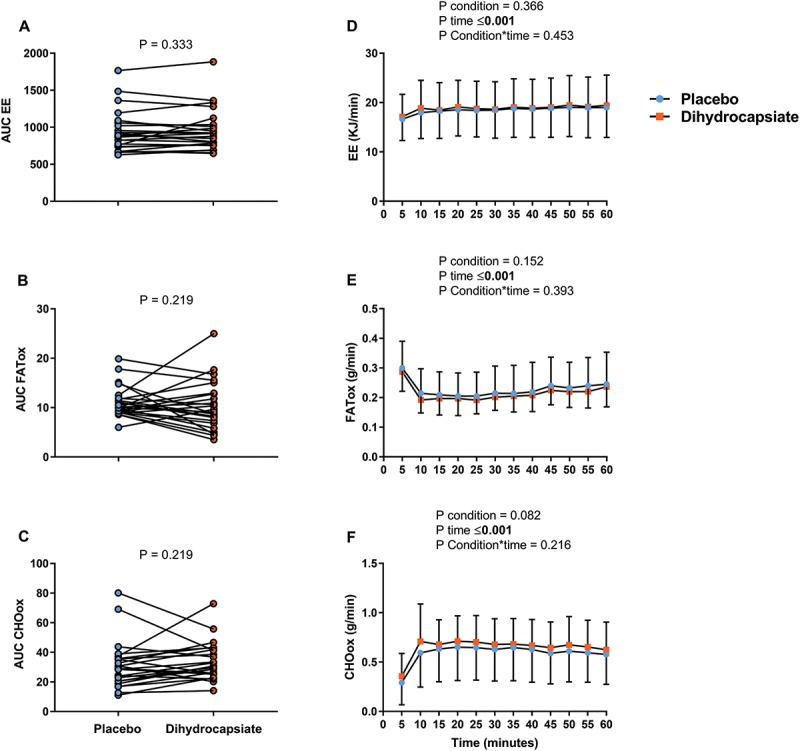
Effects of dihydrocapsiate ingestion on EE, FATox, and CHOox during aerobic exercise at FATmax intensity in men with overweight/obesity (n = 24). **Panels A, B**, and **C** show the total AUC (an indicator of the overall change) of the EE, FATox, and CHOox in the placebo vs. dihydrocapsiate conditions; P values from paired t-test comparing AUC expressed as a percentage of its baseline. **Panels D, E**, and **F** show the mean values at each time point of EE, FATox, and CHOox across these conditions; P values from linear mixed model analyses. AUC: area under the curve, CHOox: carbohydrate oxidation, EE: energy expenditure, FATox: fat oxidation. In panels D, E, and F, each single point (blue) or square (Orange) represent the mean value of each 5 min period.

No differences across conditions were found in the AUC (% baseline) of EE, FATox, and CHOox during exercise (Figure 1**; Panels A-C)** (All P ≥ 0.219). Additionally, when the AUC (% baseline) of more raw estimates (i.e. VO_2_, CO_2_, RER, or the minute ventilation (VE)) during exercise was examined, no differences were either observed across conditions **(Figure S4; Panels A-D)** (All P ≥ 0.327). The gases exchanges parameters showed that their values rapidly changed at the beginning (first 5 min) of the steady-state (effect of time, P < 0.001; Figure 1**, Panels D-F; Figure S4, Panels E-H**) but they remained stable from thereon. Neither the condition nor the interaction condition*time had a significant effect on EE, FATox, and CHOox (Figure 1**; Panels D-F)** (All P ≥ 0.216) nor on VO_2_, CO_2_, RER, and VE (**Figure S4, Panels E-H)** (All P > 0.404). Results also revealed that the ingestion of DHC had no effect on heart rate (**Fig S5, Panels A-B**) or perceived fatigue (data not shown), confirming that all participants underwent the exercise test under steady-state conditions.

### Dihydrocapsiate effects on blood parameters, skin temperature, and thermal perception during aerobic exercise at FATmax

**Figure 2.**
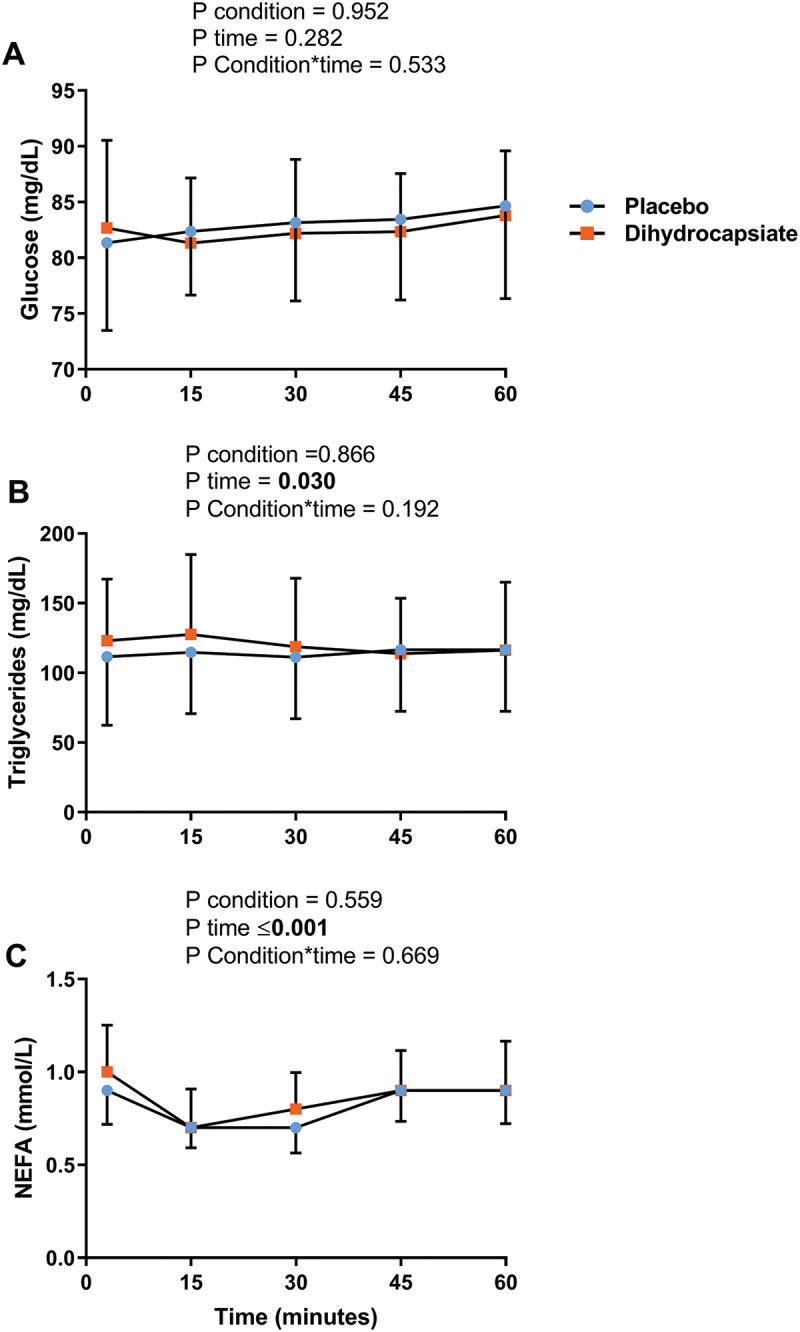
Effects of dihydrocapsiate ingestion on blood parameters during aerobic exercise at FATmax intensity in men with overweight/obesity. **Panels A, B**, and **C** respectively show the mean values at each time point of the serum levels of glucose (n = 22), triglycerides (n = 22), and NEFA (n = 16) during exercise in the placebo vs. dihydrocapsiate condition. NEFA: non-esterified fatty acids. P values from linear mixed model analyses.

**Figure 3.**
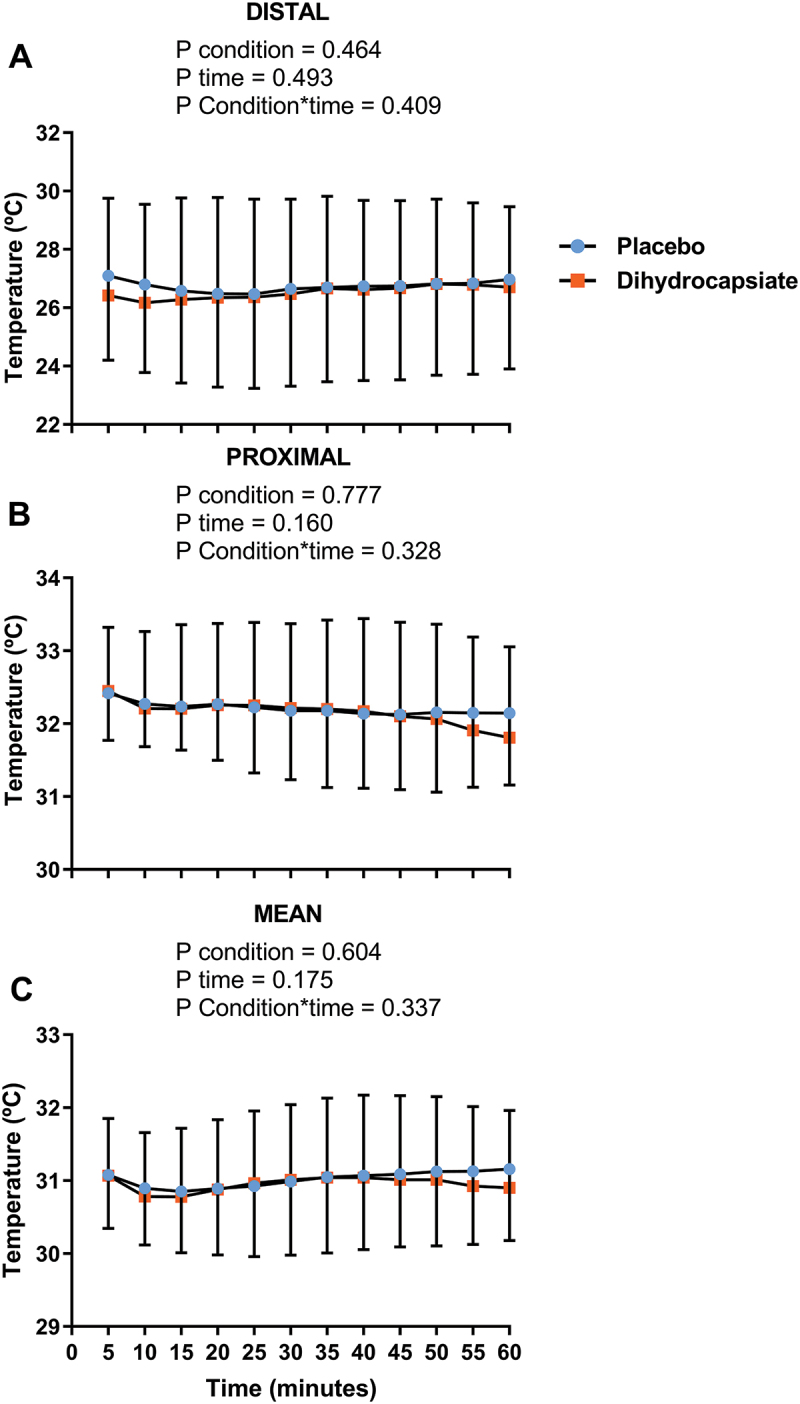
Effects of dihydrocapsiate ingestion on skin temperature during aerobic exercise at FATmax intensity in men with overweight/obesity. **Panels A, B**, and **C** respectively show the mean values at each time point of distal (n = 22), proximal (n = 17), and mean (n = 18) skin temperatures during exercise in the placebo vs. dihydrocapsiate condition. Each single point (blue) or square (Orange) represents the mean value of each 5 min period. P values from linear mixed model analyses.

**Figure 4.**
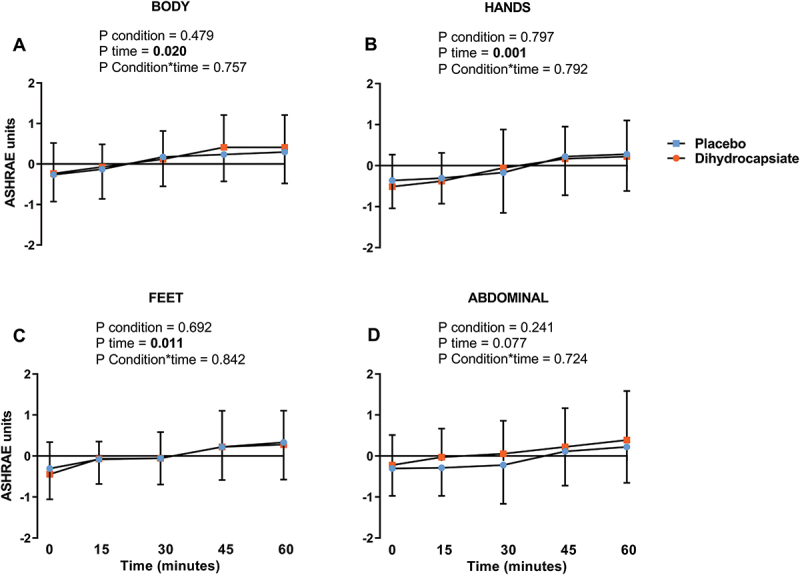
Effects of dihydrocapsiate ingestion on thermal perception during aerobic exercise at FATmax intensity in men with overweight/obesity. **Panel A, B, C**, and **D** respectively show the mean values at each time point of thermal perception on the body (n = 17), hands (n = 18), feet (n = 18), and abdominal region (n = 18). P values from linear mixed model analyses.

The ingestion of DHC did not affect serum levels of glucose, triglycerides, or NEFA during exercise (Figure 2**, Panels A-C)** (All P ≥ 0.192). Further, the mean, proximal, and distal skin temperatures during exercise were similar between DHC and placebo conditions (Figure 3**, Panels A-C)** (All P ≥ 0.328), as was the case for the thermal perception of the participants in the whole body, hands, feet, and abdominal areas (Figure 4**, Panels A-D**) (All P ≥ 0.724).

## 6. Discussion

This study investigated the acute effects of 12 mg of DHC ingestion on EE and FATox during a 60 min steady-state exercise bout at FATmax intensity in men with overweight/obesity. DHC ingestion did not increase EE or FATox as compared with placebo and had no impact on serum levels of glucose, triglycerides, or NEFA. Similarly, DHC did not affect skin temperature or temperature perception. These findings do not support the use of DHC to increase EE or FATox during aerobic exercise at FATmax intensity in men with overweight/obesity.

Our results concur with those reported by the previous study by Josse et al. [15]. In this study, they tested the ingestion of 10 mg of capsinoids, consisting of a combination of capsiate, DHC, and nordihydrocapsiate (70:23:7 ratio, respectively), in 12 healthy young sedentary and lean men (24.3 ± 3 years old, BMI = 25.5 ± 2 kg/m^2^), at rest, during 90 min of cycling at 55% at VO_2_ peak, and for 30 min of recovery. Participants ingested capsinoids 30 min before exercise. Despite the fact, they showed that capsinoids increased EE and FATox at rest, there were no significant effects of capsinoids on these parameters during exercise or recovery vs. the placebo condition [15]. Even though there were important differences regarding the participant’s characteristics and exercise protocols, the findings of Josse AR et al. agree with our results in terms of the absence of effects of capsinoids on EE, FATox, and serum NEFA levels, heart rate, and perceived fatigue during the exercise. Another study by Rossi et al. evaluated the effect of 12 mg of capsiate supplementation on EE and FATox during rest and exercise [16]. The study included a total of 30 healthy lean active men who performed a warm-up at 50% of maximal aerobic velocity for 10 minutes. Then, participants exercised at 70% of maximal aerobic velocity for 30 minutes on a treadmill, 150 min after consuming a standardized breakfast, and 35 min after consuming 12 mg of capsiate or placebo. No significant effects of capsiate on EE and FATox were reported. Of note, previous evidence has shown that capsinoids intake can enhance resting EE and FATox in humans, particularly in individuals with overweight/obesity [17] –; however, we did not observe this when capsinoids intake was combined with aerobic exercise at FATmax intensity in our study. Altogether, the supplementation with a mixture of capsinoids (capsiate, DHC, and nordihydrocapsiate) [15], purified capsiate [16], or DHC seems to have no significant effects on EE nor FATox during low-intensity aerobic exercise, neither in lean nor obese populations – which may be explained because the relative fat oxidation during this lipolytic stimuli is already high (e.g. FATmax) and overshadows any additional contribution of capsinoids. To date, the clinical potential of capsinoids to maximize the exercise benefits from a metabolic perspective is limited. Nevertheless, the role of capsinoids as ergogenic supplements in glycolytic-dependent aerobic exercise is still to be discerned.

On the other hand, and far from being consistent, the effects of capsaicinoids on energy metabolism and substrate oxidation during exercise in humans are controversial. For instance, long-distance male runners who received 10 g of hot pepper with a standard breakfast of 2.5 h before cycling for 1 h at 60% of their VO_2_max increased their CHOox both at rest and during exercise [41], while the administration of 150 mg of capsaicin in 10 untrained males 1 h before cycling during 30 min of exercise at 50% of their ventilatory threshold significantly increased their FATox [42]. Another study that evaluated the ingestion of 12 mg of capsaicin before a high-intensity intermittent bout of exercise did not find any effects on metabolic parameters, such as oxygen consumption, EE, and fatigue perception during or 20 min post-exercise in physically active men [43,44]. In line with these results, the ingestion of 1.9 mg of capsaicin and 0.7 mg of dihydrocapsaicin in 10 young healthy males that performed constant load cycling exercise time to exhaustion (TTE) trials (85% maximal work rate) did not affect substrate oxidation rates [45].

Another important fact to consider is the bioavailability of both capsinoids and capsaicinoids. Thus, while the bioavailability of orally ingested capsaicinoids in rodents is well documented, the pharmacokinetics of orally ingested capsinoids is poorly understood [46]. Orally ingested capsaicin is absorbed in the intestine and passes to the bloodstream [47], and thereby it can activate TRPV1 in peripheral muscles [48]. The fact that orally ingested capsaicin can exert its TRPV1 agonism not only within the gut but also in peripheral tissues could explain why capsaicin exerts a greater response than capsinoids in terms of ergogenic effects [48]. Opposite to capsaicinoids, there is no evidence showing that capsinoids can pass into the bloodstream in humans after being orally ingested. Plasma levels of capsinoids and their metabolite, vanillyl alcohol, were below the lower limit of quantitation after ingestion of soft gel capsules containing either 15 or 30 mg of capsinoids [49]. Nevertheless, even though capsinoids are not likely to affect EE and FATox during low-intensity exercise activities, it seems that they could have an ergogenic role in aerobic exercises that majorly relies on glycolysis/ glycogenolysis as the main source of energy, yet the evidence is scarce and uncertain [50,51]. In the light of the current evidence, the mechanisms by which capsinoids could increase EE and FATox – at least when capsinoids are orally ingested in doses below 30 mg – are likely to be explained by TRPV1 activation solely within the gastrointestinal tract [7,52]. Whether the use of higher capsinoids doses or the implementation of systems can increase the bioavailability of capsinoids, and potentially EE and FATox remain to be elucidated.

**ea**

## 7. Strengths and limitations

The main strengths of our study are: (i) the study design (i.e. randomized, triple-blinded, placebo-controlled, crossover trial); (ii) a well-phenotyped cohort of men with overweight/obesity- a population in which capsinoids could exert a greater effect in terms of increasing EE and FATox [17] – and therefore have a bigger clinical potential; and (iii) accurate monitoring of EE and FATox during exercise, including blood sampling to compare the changes of energy substrates; (iv) the fact that we employed the highest dose approved by the EFSA in order maximize the response elicited by DHC. However, our study also suffers from limitations: (i) no women were included in the trial, so we cannot have any insight into the effects of capsinoids in female participants; and (ii) our results cannot be extrapolated to those obtained with different exercise protocols (i.e. having a different exercise type, volume, or intensity).

## 8. Future research

There are no studies evaluating the effect of aerobic training interventions in combination with capsinoids supplementation on EE and FATox in humans. These studies are needed to unveil whether the chronic ingestion of capsinoids could provide additional positive effects on these variables or other health-related outcomes (e.g. weight loss or cardiometabolic health) in combination with aerobic exercise training. The inclusion of female participants in future studies is also mandatory since to date no study has evaluated the effects of capsinoids during exercise in women. As estrogen and progesterone strongly influence the physiological responses to exercise, particularly FATox could be highly influenced by sex, and thereby the effects of capsinoids in women’s FATox could be significantly different.

## 9. Conclusion

DHC supplementation does not affect energy metabolism during exercise in men with overweight/obesity. Further studies are needed to investigate if these results replicate in other populations and with other exercise types and intensities.

## Supplementary Material

Supplemental Material

## Data Availability

Data sets will be made available once the manuscript is accepted.

## Abbreviations

AUC area under the curve

BMI body mass index

CHOox carbohydrate oxidation

CVD cardiovascular disease

DHC dihydrocapsiate

EE energy expenditure

FATox fat oxidation

MFO maximal fat oxidation

RER respiratory exchange ratio

RPE rating of perceived exertion

SNS sympathetic nervous system

TRPV1 transient receptor potential vanilloid 1

VCO_2_: carbon dioxide production

VE: minute ventilation

VO_2_: volume of oxygen
