# Supplementary material for: Dihydrocapsiate does not increase energy expenditure nor fat oxidation during aerobic exercise in men with overweight/obesity: a randomized, triple-blinded, placebo-controlled, crossover trial
Source: J Int Soc Sports Nutr. 2022 Jul 19;19(1):417–36. doi: 10.1080/15502783.2022.2099757 (PMC9302013; doi:10.1080/15502783.2022.2099757)
Supplement: Supplemental Material [file RSSN_A_2099757_SM9432.docx]

**
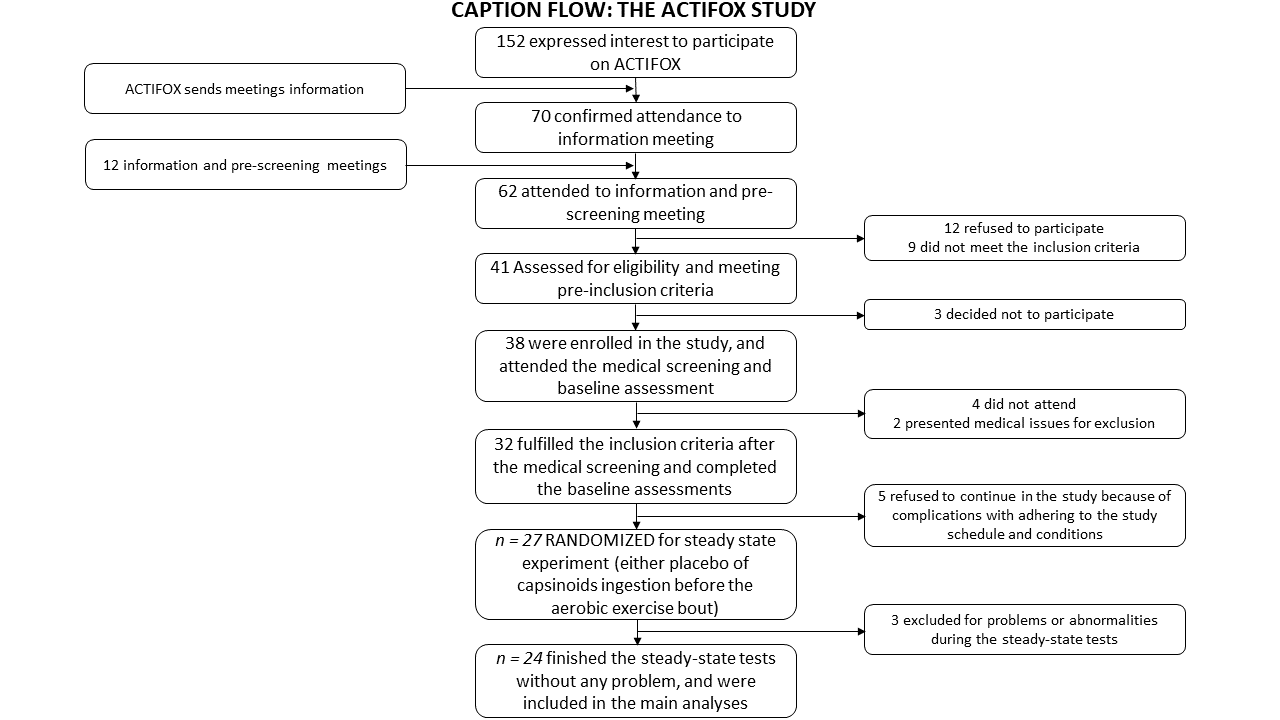
SUPPLEMENTARY INFORMATION**

**Supplementary figure 1.** Caption flow of the ACTIFOX study.


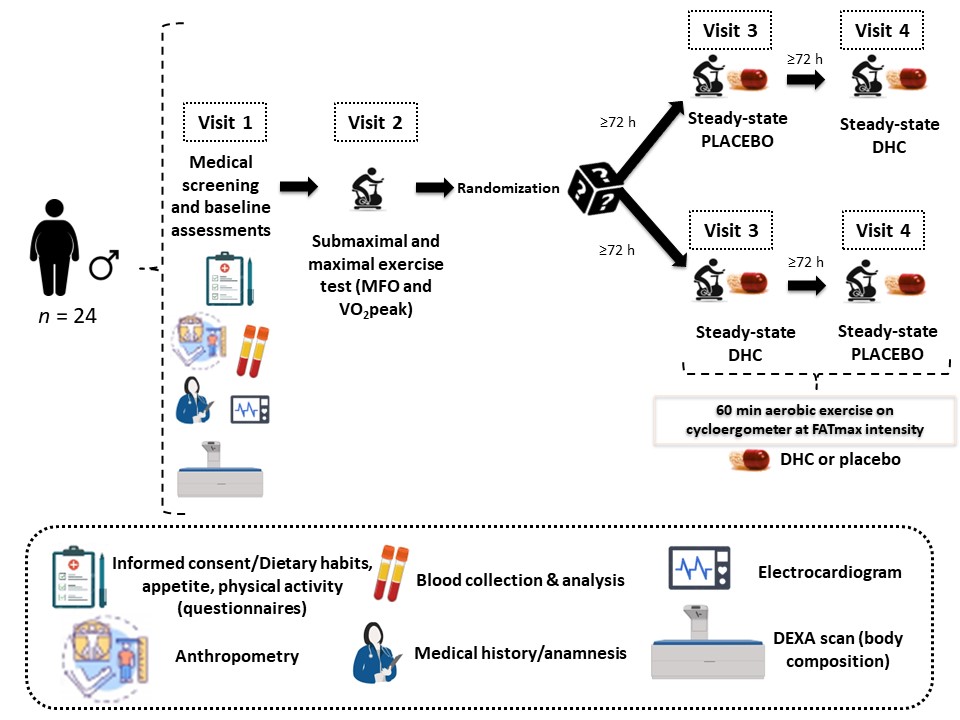


**Supplementary figure 2.** Overview of the ACTIFOX study procedures. Abbreviations: DHC: dihydrocapsiate, DEXA: dual-energy X-ray absorptiometry scan, MFO: maximal fat oxidation, VO_2_peak: peak oxygen consumption.

**
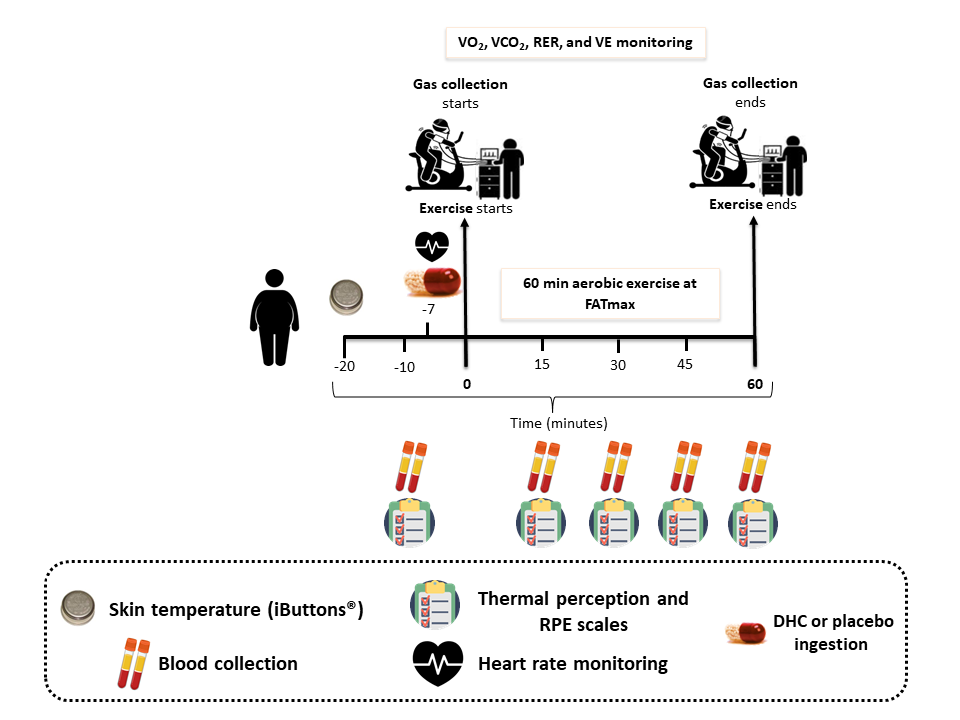
Supplementary figure 3.** Overview of the steady-state exercise bouts. Abbreviations: DHC: dihydrocapsiate, MFO: maximal fat oxidation, VO_2_peak: peak oxygen consumption. VE: minute ventilation, RER: respiratory exchange ratio, RPE: rating of perceived exertion, VCO_2_: volume of carbon dioxide production, VO_2_: volume of oxygen consumption.

**
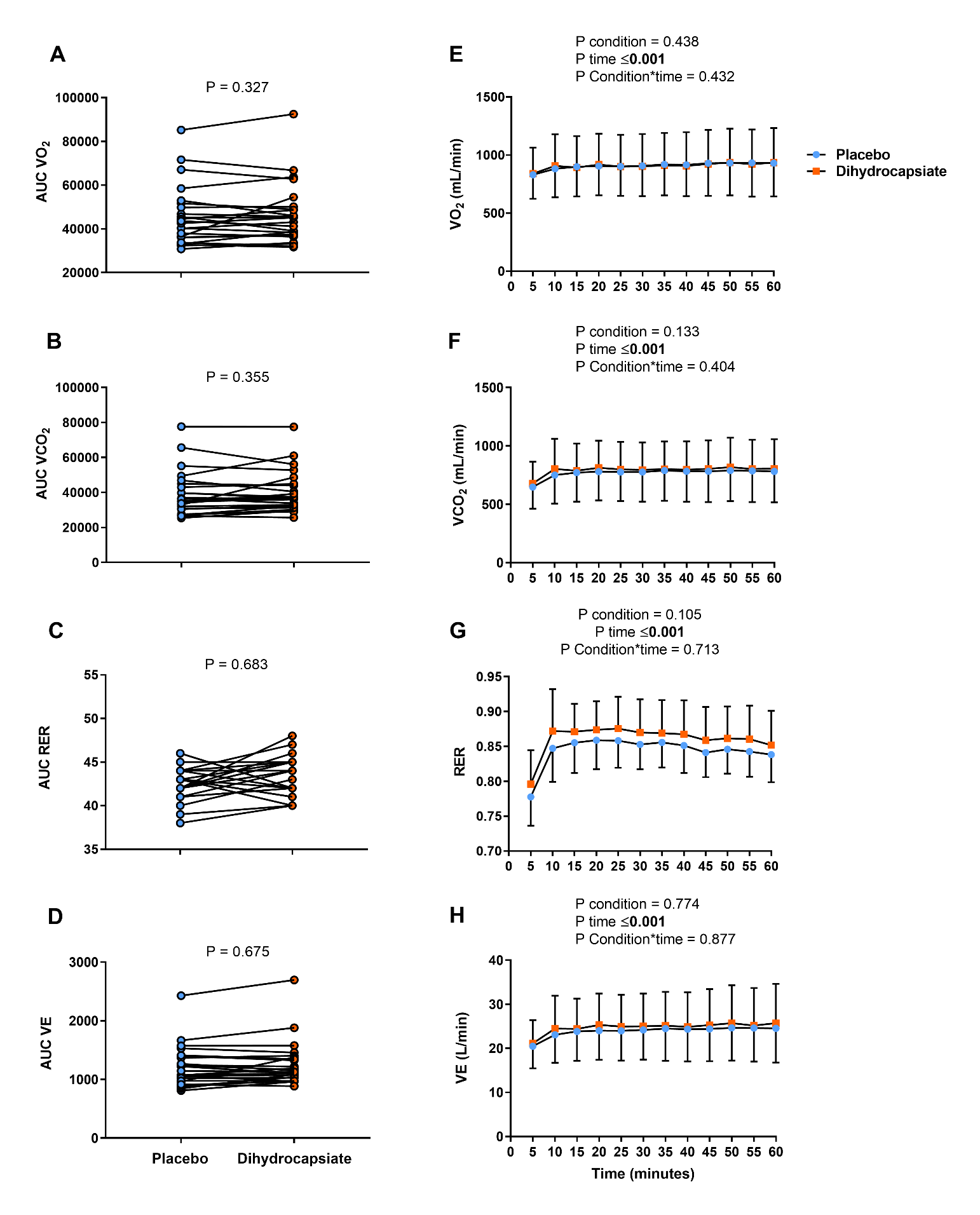
**

**Supplementary figure 4.** Effects of dihydrocapsiate on VO_2_, VCO_2_, RER, and VE during exercise in men with overweight/obesity (n=24). **Panels A, B, C,** and **D** show the total AUC of VO_2_, VCO_2_, RER, and VE in the placebo vs dihydrocapsiate conditions; P values from paired t-test comparing AUC expressed as a percentage of its baseline. **Panels E, F, G,** and **H** show the mean values at each time point of VO_2_, VCO_2_, RER, and VE across these conditions. AUC: area under the curve, VE: minute ventilation, RER: respiratory exchange ratio, VCO_2_: volume of carbon dioxide production VO_2_: volume of oxygen consumption.

**
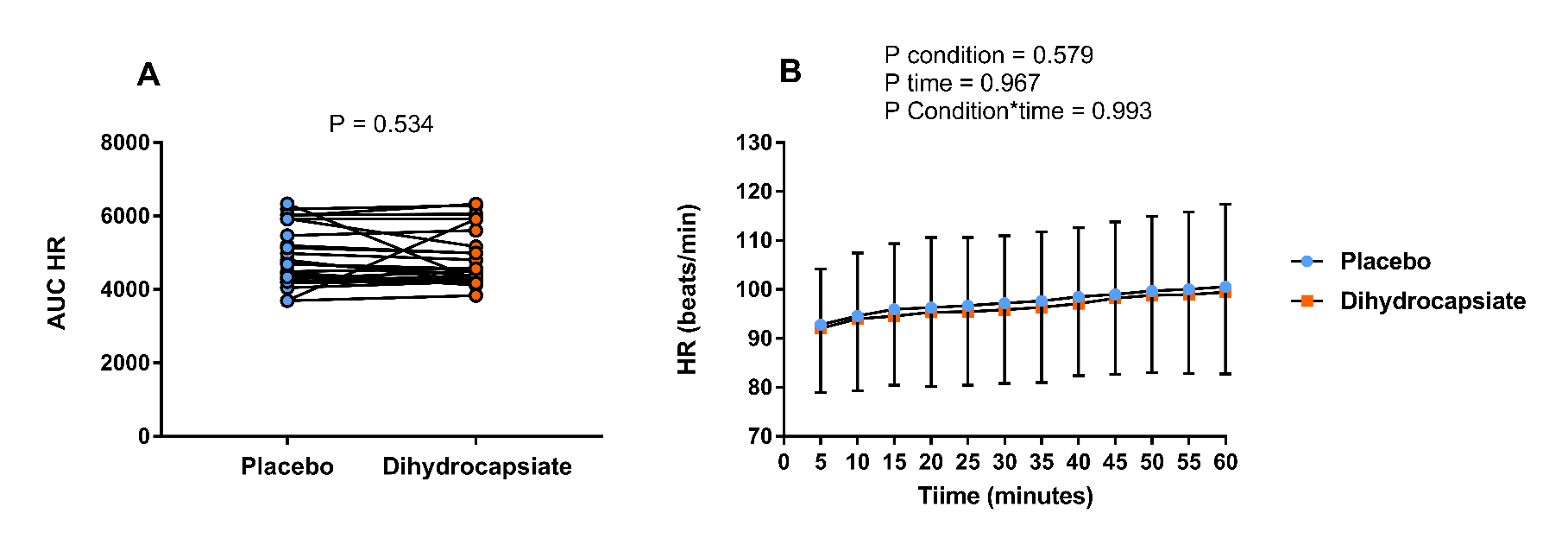
Supplementary figure 5.** Effects of dihydrocapsiate on heart rate in men with overweight/obesity (n=24). **Panel A** shows the total AUC of heart rate in placebo vs. dihydrocapsiate conditions; P-value from paired t-test comparing AUC expressed as a percentage of its baseline. **Panel B** shows the mean values at each time point of heart rate across these conditions. AUC: area under the curve.
